# Chronic productive cough in school children: prevalence and associations with asthma and environmental tobacco smoke exposure

**DOI:** 10.1186/1745-9974-2-11

**Published:** 2006-12-27

**Authors:** Edward R Carter, Jason S Debley, Gregory R Redding

**Affiliations:** 1 Department of Pediatrics, University of Washington School of Medicine, Seattle, Washington, USA

## Abstract

**Background:**

The relationships between chronic productive cough (CPC), environmental tobacco smoke (ETS) exposure, and asthma are not clearly established in children. Therefore, we wished to determine the prevalence of CPC and examine the relationships between CPC, ETS exposure, and asthma in young teenagers.

**Methods:**

We performed a cross sectional survey of 2397 Seattle middle school students, 11–15 years old, using written and video respiratory-symptom questionnaires. We defined *CPC *as – daily cough productive of phlegm for at least 3 months out of the year; *current asthma *as – *yes *to "Have you had wheezing or whistling in your chest in the past 12 months?" and* yes in the past year *to any of the four video wheezing/asthma video scenarios; and *ETS exposure *as exposed to tobacco smoke at least several hours each day. We used multilogistic  regression to examine relationships between CPC, asthma, and ETS  exposure and included in the model the potentially confounding variables race, gender, and allergic rhinitis.

**Results:**

The prevalence of CPC was 7.2%. Forty-seven percent (82/173) of children with CPC met criteria for current asthma, while only 10% (214/2224) of those without CPC had current asthma. Current asthma had the strongest associated with CPC, odds ratio (OR) 6.4 [95% CI 4.5–9.0], and ETS was independently associated with both CPC, OR 2.7 [1.8–4.1] and asthma, OR 2.7 [1.5–4.7].

**Conclusion:**

In a population of young teenagers, CPC was strongly associated with report of current asthma symptoms and also with ETS exposure. This suggests that asthma and ETS exposure may contribute to CPC in children. However, this study was not designed to determine whether asthma was the actual cause of CPC in this population of children.

## Background

Asthma is a recognized cause of persistent cough in both adults [1,2] children [3], but cough productive of sputum for more than three months out of the year, referred to as chronic productive cough (CPC), is not considered common in children with asthma. The NHLBI guidelines do not discuss productive cough as a separate sign [4], and little is known about the prevalence of CPC and its causes in children.

Chronic productive cough is a hallmark of the rare conditions cystic fibrosis, ciliary dysmotility, and bronchiectasis, but it is possible that asthma and ETS exposure lead to CPC as well. However, the relationships between asthma, ETS exposure, and CPC in children have not been delineated. Peat et al followed a cohort of school children for six years and found that the majority of those with asthma also had at some time a productive cough lasting two or more weeks, but this duration of cough was too short to be termed chronic [3]. In addition, while ETS exposure has been linked to asthma [5-8], its association with CPC, especially in children, is less clear. Lewis et al found that ETS exposure was associated with asthma symptoms but not with CPC in Alaskan native teenagers [8]. However, Janson et al surveyed young adults and identified both asthma and ETS exposure as risk factors for CPC [9].

The prevalence of CPC in a large population of children has not been well established, in part due to variations in the definition of CPC. The American Thoracic Society (ATS) defines chronic bronchitis as "cough productive of sputum for at least 3 months of the year for at least 2 years" [10], and this has become the standard for adults. However, these criteria have not been used consistently in studies of chronic cough in children. Amaral-Marques et al did use criteria that were similar to the ATS definition, and they found the prevalence of CPC in Portuguese children to be 4.9% [11]. However, they did not account for asthma or ETS exposure. Establishing the prevalence of CPC and the relationships between CPC, asthma, and ETS in children could lead to earlier diagnosis and treatment of asthma and a better understanding of the causes of CPC.

In 2003 we participated in Phase III of the International Study of Allergies and Asthma in Childhood (ISAAC) as part of an effort to determine the prevalence of asthma symptoms in children throughout the world [12,13]. Seattle middle-school students completed written and video respiratory-symptom surveys. We added questions on ETS exposure and CPC in order to determine the prevalence of CPC and examine the relationships between CPC, asthma, and ETS exposure. Some of the results of this study have been published in abstract form [14].

## Methods

### Subjects

In June 2003, students from the six middle schools in Seattle, Washington that participated in the ISAAC Phase III study were asked to complete written and video respiratory-symptom questionnaires. The Seattle School Board and The University of Washington Human Subjects Committee approved the protocol and waived written informed consent. We provided detailed written information to parents as well as verbal and written information to the students, and gave them ample time to refuse participation. We targeted children in the 7^th ^and 8^th ^grades, but 6^th ^grade students were also eligible. Investigators oversaw completion of the questionnaires during typical class periods. Eighty-six percent (2397/2797) of the eligible students completed the questionnaires. School absenteeism accounted for the vast majority of students who did not complete surveys.

### Study questionnaire and administration

The written survey contained core ISAAC questions on asthma, allergic rhinitis, and eczema, and we added questions on CPC and tobacco smoke exposure. In addition to the written questionnaire, students viewed the international version of the ISAAC video, which has one cough and four wheezing scenarios depicting children with signs of asthma.

### Definitions

#### CPC

required positive responses to both of the written questions, "Have you had a daily cough as often as 3 months out of the year?" and "Do you bring up phlegm, sputum, or mucous from your lungs as often as 3 months out of the year?" The personnel assisting with the study asked students if they knew what was meant by "sputum/phlegm", and if there was any confusion then they provided explanations. We did not use responses to the cough video question because this scenario showed a child with a non-productive hacking cough.

#### ETS exposure

The ETS question "How much time do you think that you spend around tobacco smoke?" had three possible responses – never or very little, occasionally, and several hours a day. Students were categorized has having ETS exposure if they answered "several hours a day."

#### Current asthma

required a positive response to the written question, "Have you had wheezing or whistling in your chest in the past 12 months?" and a "yes in the past year" to any of the four video wheezing/asthma video scenarios. We did not use responses from the cough video scenario as part of the diagnostic criteria for asthma because we felt that this scenario was not representative enough of asthma. The current asthma group included both patients with and without a physician diagnosis of asthma.

#### No asthma

required an answer of *no *to wheezing in the past year, *no *to a physician diagnosis of asthma, and* no in the past year *to all four of the video wheezing scenarios.

#### Possible asthma

students who did not fit into either the current asthma or no asthma groups. These students had some positive responses to asthma questions but did not meet our specific criteria for current asthma.

#### Allergic rhinitis

Allergic rhinitis is a common cause of post nasal drip and cough with a high prevalence in patients with asthma. Thus, we wished to identify students who might have allergic rhinitis. We classified students as having allergic rhinitis if they answered *yes *to either of the two ISAAC questions, "Have you ever had a problem with sneezing, or a runny, or blocked, or stuffy nose when you did not have a cold or flu, that was accompanied by itchy-watery eyes?" or "Have you ever had hay fever?" These ISAAC questions have been validated and have a high specificity for atopy confirmed by skin testing [15].

### Outcomes and statistical analysis

The primary objectives were to establish the prevalence of CPC in this population and to determine the associations of CPC with current asthma symptoms and ETS exposure. We also examined the relationship between ETS exposure and asthma. Demographic data were characterized using descriptive statistics, and differences between groups were analyzed with Chi Square. Using SPSS 11.5, we performed univariate analysis followed by multivariable logistic regression to assess independent associations between current asthma, ETS exposure and CPC. We included the potential confounding variables of allergic rhinitis, gender, and race in our model. We explored potential effect modification by adding the following multiplicative interaction terms to each model: gender × ETS exposure, gender × current asthma, ETS exposure × current asthma, race × ETS exposure, and race × current asthma. We did not include any multiplicative terms in our final regression model because we found no evidence of effect modification. We expressed these relationships as odds ratios (OR) with their respective 95% confidence intervals [95% CI].

## Results

The demographics of the students are denoted in Table 1. The median age of the students was 13 years, and most of them were Caucasian, African American, or Asian. The prevalence of CPC was 7.2% (173/2397), and the prevalence rates of current asthma and ETS exposure were 12.4% and 9.3%, respectively. Of those students with CPC, 34% stated their sputum was white or clear, 47% reported it was yellow, and 15% claimed that it was green. Compared to the total group, a higher proportion of students reporting CPC were girls (63% vs. 50%, p = 0.024). Similarly, a higher proportion of children with current asthma were girls (59% vs. 52%, p = 0.024). Five percent of the students claimed to have smoked at least one cigarette in the past month. However, only 30% of the students actually answered this question, a response rate too low to accurately assess the effects of active smoking. Report of allergic rhinitis was also much more common in children with CPC (Table 2).

**Table 1:** Demographics of middle-school children with CPC*

Characteristic	Children with CPC (n = 173)	Children without CPC (n = 2224)
Prevalence in population	7.2% [3.3–11.1]	--
Age (years): median (range)	13 (12–15)	13 (11–16)
% Female	63% [56–70]	50% [48–52]
Race (%)		
Caucasian	29% [22–36]	31% [29–33]
African American	27% [20–34]	20% [18–21]
Asian	23% [17–29]	32% [30–34]
Native American	4% [1–7]	2% [1–3]
Other	17% [11–23]	15% [13–16]

* See text for the definition of chronic productive cough (CPC). Values in brackets are 95% confidence intervals.

**Table 2:** Adjusted multivariate associations of asthma and ETS exposure with CPC

Condition	Students with CPC N = 173	Students without CPC N = 2224	Odds Ratio [95% CI]
Current Asthma	82 (47%)*	214 (10%)	5.2 [3.6–7.5]
ETS exposure	43 (25%)	180 (8%)	2.9 [1.4–9.4]
Allergic Rhinitis	103 (60%)	549 (25%)	2.6 [1.9–3.8]
Female gender	108 (62%)	1108 (50%)	1.5 [1.0–2.1]

*The numbers in parentheses are the percent of students in each group who have the condition; i.e., 82/173 (47%) of children with CPC had current asthma.

**Figure 1:**
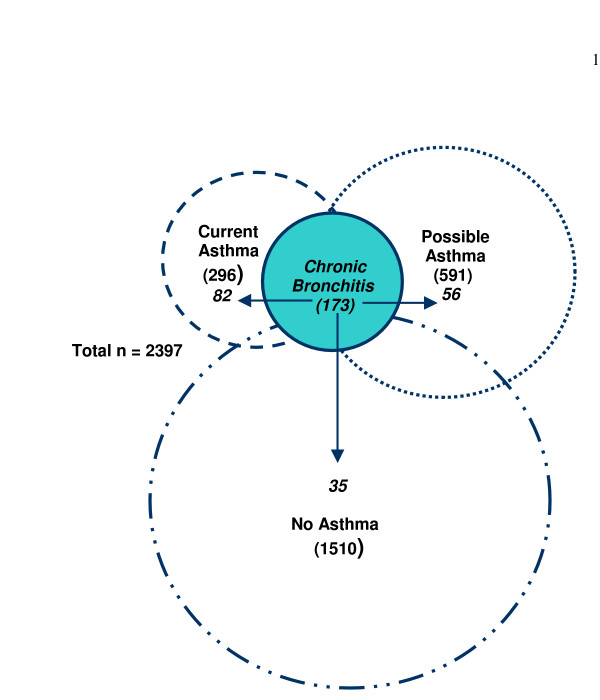
**The relationship between CPC and asthma**. This Venn diagram depicts the relationship between chronic productive cough (CPC) and asthma. See text for definitions of the current, possible, and no asthma groups. While the criteria for these groups make them mutually exclusive, they are shown as overlapping to indicate that in reality there is crossover between the groups. The numbers in parentheses are the number of children with the condition, e.g. current asthma. The proportions of children in each group with CPC (28%, 9.5%, and 2.3% for the current asthma, possible asthma, and no asthma groups, respectively), were statistically significantly different, p < 0.001 for each comparison.

Current asthma was strongly associated with CPC (OR by univariate analysis 6.4 [4.5–9.0]). The association remained strong after accounting for interactions of gender, ETS exposure, and allergic rhinitis in a multilogistic regression model (Table 2). Children with CPC were five times more likely to have current asthma than those without CPC. Nearly half of the children with CPC (82/173) had current asthma compared to only 10% (214/2224) of those without CPC (Table 2). There were 296 children with current asthma, 1510 that met criteria for no asthma, and 591 children with possible asthma. Of the 173 children with CPC, 138 had current asthma or possible asthma, while only 35 met criteria for no asthma (Figure 1). Twenty-eight percent (82/296) of the current asthma group reported CPC compared to 9.5% (56/591) of the children with possible asthma and only 2.3% (35/1510) of the no asthma group (Figure 1).

Environmental tobacco smoke exposure was associated with CPC by univariate analysis (OR 2.7 [1.8–4.1]), and this association remained similar in the multilogistic regression analysis (Table 2). Environmental tobacco smoke exposure was also associated with current asthma, OR 2.7 [1.5–4.7]. Nineteen percent of the children (43/223) with ETS exposure had CPC compared to 6% (130/2174) of those without ETS exposure, p < 0.01. Twenty-three percent (52/223) of the children with ETS exposure had current asthma, while only 8% of those without current asthma reported ETS exposure, p < 0.01. More children with both asthma and CPC reported ETS exposure than did the children with asthma but no CPC; 33% (27/82) vs. 12% (25/214), p < 0.01.

## Discussion

In this survey-based study of almost 3000 middle-school students, the prevalence of CPC, defined as a daily cough productive of phlegm for at least three months out of the year, was 7.2%. Report of current asthma symptoms was strongly associated with CPC, even after correcting for allergic rhinitis, and almost half of the children with CPC met criteria for current asthma. Environmental tobacco smoke exposure was also independently associated with CPC. These findings suggest that CPC can be a manifestation of asthma and that asthma should be considered in the differential diagnosis of children who present with a CPC.

It is important to establish what is meant by CPC, often referred to as chronic bronchitis. In the 1950s, the British Medical Research Council defined chronic bronchitis as "cough productive of sputum for at least 3 months of the year for at least 2 years without an identifiable cause [16], and the ATS adopted this definition in 1962 [10]. While many investigations of CPC in young adults and children have employed similar definitions [11,17-20], others have not [8,9]. Consequently, this makes it difficult to compare the prevalence and causes of CPC across studies. In addition, while chronic bronchitis is a term that is inherently linked to CPC, it has many connotations. Taussig et al noted that only 55% of pediatricians and 74% of family practitioners surveyed considered CPC lasting at least 3 months of the year important in diagnosing chronic bronchitis [21], and Bobadilla et al found that only a minority of patients with physician-diagnosed chronic bronchitis actually met ATS criteria [22]. Thus, it is more precise to use the descriptive term CPC in lieu of the label 'chronic bronchitis'. The ATS definition was established primarily for adults, and there are causes of CPC in adults that are much less common in children, including active cigarette smoking and chronic obstructive pulmonary disease. Nevertheless, by adopting standard criteria for CPC, it will be possible to compare results across studies as well as age groups.

Our study is one of the few to establish the prevalence of CPC in a large population of children using an adaptation of the ATS criteria. Our criteria only differed from the ATS criteria in that we required cough productive for sputum over one year rather than in two consecutive years. We found the prevalence of CPC to be 7.2%, which is higher than the 4.9% prevalence noted by Amaral-Marques et al in 4148 Portuguese school-aged children [11]. Their prevalence may have been lower because they required productive cough in two consecutive years. As with our study, they observed that a higher proportion (62%) of the young teenagers with CPC were girls. One possible explanation for this female predominance is that asthma and CPC are closely linked, and, as noted in our study as well as others [23,24], more teenagers with asthma are girls. Girls did not report significantly more ETS exposure, so it is unlikely that ETS exposure was a factor in the female predominance of CPC.

Investigators have surveyed of young adult populations to determine the relationships between smoking, CPC, and asthma, but there are few data in children. Cerevi et al identified active cigarette smoking to be the primary risk factor for CPC in young adults [18]. However, almost 20% of their subjects with CPC had asthma and approximately 30% were non-smokers. Compared to the active smokers, the non-smokers were younger and were more likely to be female and to have asthma. Janson et al in a survey of 18,277 young adults noted a positive, albeit weak, association of CPC with ETS exposure and a stronger association with asthma [9]. However, Lewis et al noted that ETS exposure was a risk factor for asthma but not for CPC in Alaska native teenagers [8]. Environmental tobacco smoke exposure has been linked to asthma exacerbations [5-7], but the association of ETS exposure with persistent asthma symptoms is less well established. While our study was not designed to determine the causes of CPC, our results suggest that asthma and ETS exposure independently increase the risk of having CPC. Furthermore, the fact that 28% of the children with current asthma reported CPC indicates that CPC may be a more frequent complaint in patients with asthma than previously recognized.

There are rare conditions, e.g. cystic fibrosis, that frequently present with CPC, but the most common causes of CPC have not been determined on a population level. However, the causes of CPC have been studied in select pediatric populations. Seear et al determined the causes of CPC in a group of children specifically referred for evaluation of that complaint [20]. They found that of 81 children presenting with "a productive or rattly cough, with or without wheezing, on most days for 3 consecutive months or more", 14 had probable asthma and 33 had other conditions that explained their cough. However, there were 34 children in whom there were no clear etiologies, and they were labeled as having chronic bronchitis. Of note, eight of these children (24%) were Native American. The authors postulated that lower respiratory tract infections early in life resulted in lung damage/inflammation and a propensity towards chronic cough. Native Americans appear to be prone to CPC. Lewis et al found that 30% of Alaskan native teenagers reported CPC, many of whom did not meet criteria for current asthma symptoms [8]. This population has an unusually high prevalence of bronchiectasis, presumably due to a predilection to damage from lower respiratory tract infections [25]. In our study, only 4% of the students claimed Native American heritage, and it is unlikely that bronchiectasis accounted for many of the cases of CPC. Marchant et al evaluated 108 children referred to a pediatric respiratory practice for assessment of cough of > 3 weeks duration [26]. The mean age was 2.6 years and 89% had wet cough. They found the most common diagnosis to be protracted bacterial bronchitis, based on a positive culture of bronchoalveolar lavage fluid and response to antibiotic treatment. Fewer than 5% had asthma as the primary diagnosis. The studies by Seear et al and Marchant et al suggest that children referred to a respiratory clinic for evaluation of cough often have diagnoses other than asthma. However, it is likely that many of the patients with asthma that have CPC are not referred to specialists, and these studies were not designed to assess the frequency of CPC in children with asthma.

Allergic rhinitis is a common cause of post nasal drip and chronic cough, and we found that report of allergic rhinitis was associated with CPC. However, using a multilogistic regression model that included allergic rhinitis as a co-variate, we found that current asthma had the strongest independent association with CPC. Nevertheless, we cannot rule out the possibility that allergic rhinitis was the cause of CPC in some of the children who also reported current asthma symptoms.

There were limitations to our study. This was a cross-sectional study, and we did not follow the children longitudinally. The results were based on self-reports, and we did not use physical examinations or tests to confirm the diagnosis of asthma or identify other potential causes of cough. Therefore, the children that reported CPC and also met criteria for asthma and/or ETS exposure may have had other causes for their cough, including allergic rhinitis, chronic sinusitis, or the rarer diseases cystic fibrosis and bronchiectasis. Asthma is unlikely to be the cause of CPC in patients with purulent sputum, and only 15% of the students in our study reported green sputum. Thus, it is important to evaluate children who have cough productive of purulent sputum for other conditions even if they have asthma. The questions used to define CPC, while standard, have not been validated in children. Young teenagers may have difficulty recalling their symptoms over a year's time and understanding what is meant by sputum or phlegm production. The study personnel that administered the surveys to the students were available to explain the questions to the students, so we believe that most of the students were capable of answering the questions. Nevertheless, the results of our study should be interpreted with caution due to the lack of physician-confirmation of CPC, asthma, and other respiratory conditions in the respondents. Our criteria for current asthma, which we have used previously [13], were designed to have a fairly high specificity at the risk of decreased sensitivity [27] and likely resulted in the misclassification of some of the subjects. The prevalence of ETS exposure in our patient population was lower than that reported in other studies [28,29], possibly due to the reliance on self report and the requirement of being around cigarette smoke for at least several hours each day. However, the prevalence of ETS exposure in the students reporting chronic productive cough (25%) was similar to the prevalence of ETS exposure in homes reported by both Soliman et al and Sexton et al [28,29]. Finally, we could not assess active cigarette smoking as too few students responded to that question. Only 5% of the students in our study reported having smoked at least one cigarette within the past month compared to 9.6% of 3379 8^th^-graders who responded to this question on the 2004 Washington State Healthy Youth Survey [30]. Therefore, some of the effects attributed to ETS exposure may have been due to active smoking.

Asthma is a common cause of cough but not necessarily of CPC. We found an association of self-reported CPC and asthma symptoms. However, this does not prove that the CPC was due to asthma nor does it suggest that children with asthma and CPC do not require evaluation for other causes of CPC, especially in those with purulent sputum. However, the strong association between asthma symptoms and CPC identified in our study suggests that CPC may be more common in children with asthma than previously thought.

## Conclusion

The prevalence of CPC in young teenagers, based on self-report of cough productive of phlegm for at least 3 months out of the year, is approximately seven percent. Children with CPC and/or ETS exposure are more likely to report asthma symptoms. We found that CPC was more common in children with current asthma symptoms and/or ETS exposure than in children without those conditions. Furthermore, current asthma and ETS exposure were strongly and independently associated with CPC. In this limited epidemiological study children reporting CPC had an increased risk of asthma symptoms. However, asthma is unlikely to be the cause chronic cough productive of purulent sputum, and patients with purulent sputum should be evaluated for other conditions even if they have asthma. Further studies are needed to determine the frequency of CPC in children who have confirmed asthma and to establish whether children presenting with CPC truly have asthma as the cause of their CPC *vs*. other underlying diseases.

## Abbreviations

ATS – American Thoracic Society

CPC – Chronic productive cough

ETS – Environmental tobacco smoke

ISAAC – International Study of Allergies and Asthma in Childhood

OR – odds ratio

## References

[B1] Irwin RS, Corrao WM, Pratter MR (1981). Chronic persistent cough in the adult: the spectrum and frequency of causes and successful outcome of specific therapy. Am Rev Respir Dis.

[B2] Irwin RS, Curley FJ, French CL (1990). Chronic cough. The spectrum and frequency of causes, key components of the diagnostic evaluation, and outcome of specific therapy. Am Rev Respir Dis.

[B3] Peat JK, Woolcock AJ, Leeder SR, Blackburn CRB (1980). Asthma and bronchitis in Sydney schoolchildren. 1. Prevalence during a six-year study. Am J Epidemio.

[B4] (1997). NHL National Heart Lung and Blood Institute Guidelines for the Diagnosis and management of Asthma: Expert Panel Report No. 2. NIH Publication No 97-4051.

[B5] Cunningham J, O'Connor GT, Dockery DW, Speizer FE (1996). Environmental tobacco smoke, wheezing, and asthma in children in 24 communities. Am J Respir Crit Care Med.

[B6] Evans D, Levison MJ, Feldman CH, Clark NM, Wasilewski Y, Levin B, Mellins RB (1987). The impact of passive smoking on emergency room visits of urban children with asthma. Am Rev Respir Dis.

[B7] Chilmonczyk BA, Salmun LM, Megathlin KN, Neveux LM, Palomaki GE, Knight GJ, Pulkkinen AJ, Haddow JE (1993). Association between exposure to environmental tobacco smoke and exacerbations of asthma in children. N Engl J Med.

[B8] Lewis TC, Stout JW, Martinez S, Morray B, White LC, Heckbert SR, Redding GR (2004). Prevalence of asthma and chronic respiratory symptoms among Alaska native children. Chest.

[B9] Janson C, Chinn S, Jarvis D, Burney P (2001). Determinants of cough in young adults participating in the European Community Respiratory Health Survey. Eur Respir J.

[B10] (1962). Definitions and classification of chronic bronchitis, asthma, and pulmonary emphysema: American Thoracic Society. Am Rev Respir Dis.

[B11] Amaral-Marques R, Cochito ML, Cruz M, Villar M, Villar TG (1980). Prevalence of chronic bronchitis in the children of Lisbon. Bronchopneumologie.

[B12] Ellwood P, Asher MI, Beasley R, Clayton TO, Stewart AW (2005). ISAAC Steering Committee. The International Study of Allergies and Asthma in Childhood (ISAAC): Phase three rationale and methods. Int J Tuberc Lung Dis.

[B13] Carter ER, Debley JS, Redding GR (2005). Changes in asthma prevalence and impact on health and function in Seattle middle-school children: 1995 versus 2003. Ann Allergy Asthma Immunol.

[B14] Carter ER, Debley JS, Redding GR (2004). Chronic bronchitis in children: How much is due to asthma?. Chest.

[B15] Braun-Fahrlander C, Wuthrich B, Gassner M, Grize L, Sennhauser FH, Varonier HS, Vuille JC (1997). Validation of a rhinitis symptom questionnaire (ISAAC core questions) in a population of Swiss school children visiting the school health services. SCARPOL-team. Swiss Study on Childhood Allergy and Respiratory Symptom with Respect to Air Pollution and Climate. International Study of Asthma and Allergies in Childhood. Pediatr Allergy Immunol.

[B16] Fletcher CM, Elmes PC, Fairbairn AS, Wood CH (1959). The significance of respiratory symptoms and the diagnosis of chronic bronchitis in a working population. Br Med J.

[B17] Cerveri I, Accordini S, Verlato G, Corsico A, Zoia MC, Casali L, Burney P, de Marco R, for the European Community Respiratory Health (ECRHS) Study Group (2001). Variations in the prevalence across countries of chronic bronchitis and smoking habits in young adults. Eur Respir J.

[B18] Cerveri I, Accordini S, Corsico A, Zoia MC, Carrozzi L, Cazzoletti L, Beccaria M, Marinoni A, Viegi G, de Marco R, for the ISAYA Study Group (2003). Chronic cough and phlegm in young adults. Eur Respir J.

[B19] Pallasaho P, Lundbäck B, Meren M, Kiviloog J, Loit H-M, Larsson K, Latinen LA (2002). Prevalence and risk factors for asthma and chronic bronchitis in the capitals Helsinki, Stockholm, and Tallinn. Respir Med.

[B20] Seear M, Wensley D (1997). Chronic cough and wheeze in children: do they all have asthma?. Eur Respir J.

[B21] Taussig LM, Smith SM, Blumenfeld R (1981). Chronic bronchitis in childhood: what is it?. Pediatrics.

[B22] Bobadilla A, Guerra S, Sherrill D, Barbee R (2002). How accurate is the self-reported diagnosis of chronic bronchitis?. Chest.

[B23] Debley JS, Redding GJ, Critchlow CW (2004). Impact of adolescence and gender on asthma hospitalization: a population-based birth cohort study. Pediatr Pulmonol.

[B24] Venn A, Lewis S, Cooper M, Hill J, Britton J (1998). Questionnaire study of effect of sex and age on the prevalence of wheeze and asthma in adolescence. Br Med J.

[B25] Singleton R, Morris A, Redding G, Poll J, Holck P, Martinez P, Kruse D, Bulkow LR, Petersen KM, Lewis C (2000). Bronchiectasis in Alaska native children: causes and clinical courses. Pediatr Pulmonol.

[B26] Marchant JM, Masters B, Taylor SM, Cox NC, Seymour GJ, Chang AB (2006). Evaluation and outcome of young children with chronic cough. Chest.

[B27] Gibson PG, Henry R, Shah S, Toneguzzi R, Francis JL, Norzila MZ, Davies H (2000). Validation of the ISAAC video questionnaire (AVQ3.0) in adolescents from a mixed ethnic background. Clin Exp Allergy.

[B28] Soliman S, Pollack H, Warner K (2004). Decrease in the prevalence of environmental tobacco smoke exposure in the home during the 1990s in families with children. Am J Public Health.

[B29] Sexton K, Adgate JL, Church TR, Hecht SS, Ramachandran G, Greaves IA, Fredrickson AL, Ryan AD, Carmella SG, Geisser MS (2004). Children's exposure to environmental tobacco smoke: using diverse metrics to document ethnic/racial differences. Environ Health Perspect.

[B30] The RMC Research Corporation, the Washington State Healthy Youth Survey 2004 Analytic Report. http://www3.doh.wa.gov/HYS/ASPX/HYSQuery.aspx.

